# Accelerometer data from the performance of sit-to-stand test by elderly people

**DOI:** 10.1016/j.dib.2020.106328

**Published:** 2020-09-23

**Authors:** Diogo Luís Marques, Henrique Pereira Neiva, Ivan Miguel Pires, Daniel Almeida Marinho, Mário Cardoso Marques

**Affiliations:** a Department of Sport Sciences, University of Beira Interior, Covilhã, Portugal; b Instituto de Telecomunicações, Universidade da Beira Interior, Covilhã, Portugal; c Computer Science Department, Polytechnic Institute of Viseu, Viseu, Portugal; d Research Center in Sports Sciences, Health Sciences and Human Development, CIDESD, Covilhã, Portugal

**Keywords:** Health, Sit-to-Stand Test, Sensors, Accelerometer, Mobile devices, Neuromuscular function, Older adults

## Abstract

The sit-to-stand test is commonly used by clinicians and researchers to analyze the functional capacity of older adults. The test consists to stand up and sit down from a chair and can be applied either in function of a predetermined number of repetitions to be completed or according to a specific time. The most common tool used by the evaluators is the chronometer, due to its low cost and ease of use. However, this tool may miss some important data throughout the test, such as the stand-up time and the total time of each repetition, as well as other kinematic and kinetic variables. Therefore, it is necessary to develop new cheap and affordable tools to capture these data with reliability. In this perspective, the development of mobile applications can be a valid and reliable alternative for the automatic calculation of different variables with sensors’ data, including acceleration, velocity, force, power, and others. Thus, in this paper, we present a dataset related to the acquisition of the accelerometer data from a commodity smartphone for the measurement of different variables during the sit-to-stand test with institutionalized older adults. Forty participants (20 men and 20 women, 78.9 ± 8.6 years old, 71.7 ± 15.0 kg, 1.57 ± 0.1 m) from five community-dwelling centers (Centro de Dia e Apoio Domiciliário de Alcongosta, Lar Nossa Senhora de Fátima, Centro Comunitário das Minas da Panasqueira, Lar da Misericórdia, and Lar da Aldeia de Joanes) from Fundão, in Portugal, volunteered to participate in the data acquisition. A mobile phone was attached to the waist of the participants to capture the data during the sit-to-stand test. Then, seated in an armless chair with the arms crossed over the chest, the participants stood up and sat down in a chair six times. The stand-up action was ordered by an acoustic signal emitted by the mobile application. All data were acquired with the mobile application, and the outcome measures were the reaction time, total time, stand-up time and movement time. This paper describes the procedures to acquire the data. These data can be reused for testing machine learning or other methods for the evaluation of neuromuscular function in older adults during the sit-to-stand test.

## Specifications Table

**Table:** 

Subject	Orthopaedics, Sports Medicine and Rehabilitation
Specific subject area	Sit-to-Stand Test Physical therapy Sports Elderly
Type of data	Table; Chart
How data were acquired	A Smartphone XIAOMI MI A1 was used in a wristband. It acquired the accelerometer data with an Android application installed.
Data format	Raw text files
Parameters for data collection	Older adults from the same institution, but different community-dwelling centers received the explanation about the sit-to-stand test. Next, they were instrumented with the mobile device in a waistband. The equipment was a simple smartphone, and the data acquired from the accelerometer sensor was stored in the mobile device's storage for further analysis.
Description of data collection	After the instrumentation, the participants were seated on a chair. When they heard the acoustic signal emitted by the mobile application, they performed the sit-to-stand movement one time. The task was repeated six times, with a 15-s rest between repetitions. The data was acquired from the tri-axial accelerometer of the mobile device. The accelerometer model embedded in the mobile device is the Bosch BMI120, and the frequencies of data acquisition are 200 Hz/400 Hz for specific forces, and 100 Hz/400 Hz for angular velocities.
Data source location	Primary data sources: Institution: Centro de Dia e Apoio Domiciliário de Alcongosta City/Town/Region: Alcongosta Country: Portugal Latitude and longitude for collected samples/data: 40° 6’ 56.682” N 7° 29’ 1.398” W Institution: Lar Nossa Senhora de Fátima City/Town/Region: Fundão Country: Portugal Latitude and longitude for collected samples/data: 40° 8’ 12.827” N 7° 30’ 4.3” W Institution: Centro Comunitário das Minas da Panasqueira City/Town/Region: Minas da Panasqueira
	Country: Portugal Latitude and longitude for collected samples/data: 40° 9’ 5.45” N 7° 44’ 33.599” W Institution: Lar da Misericórdia da Santa Casa da Misericórdia do Fundão City/Town/Region: Fundão Country: Portugal Latitude and longitude for collected samples/data: 40° 8’ 8.893” N 7° 30’ 28.702” W Institution: Lar da Aldeia de Joanes da Santa Casa da Misericórdia do Fundão City/Town/Region: Aldeia de Joanes Country: Portugal Latitude and longitude for collected samples/data: 40° 8’ 9.179” N 7° 31’ 6.825” W
Data accessibility	Repository name: Sit-to-Stand Test Collected with Elderly People From Centre of Portugal Data identification number: 10.17632/335rmgrfw2.4 Direct URL to data: https://data.mendeley.com/datasets/335rmgrfw2/4

## Value of the Data

• These data is useful to predict falls and other diseases in older adults [1,2].
• These data may help to detect changes in muscle power before and after resistance training [3], [4], [5], [6].
• These data can be processed to identify the sit-to-stand movement, and the different effects related to training and detraining on neuromuscular function in older adults can be identified [7,8].
• The data available in the dataset can be used to analyze the kinematic and kinetic parameters during the sit-to-stand test when performed by older adults [9], [10], [11].

## 1 Data Description

The data reported in this article is related to the performance of the Sit-to-Stand test by older adults from Fundão municipality. A smartphone was used to acquire the accelerometer data during the performance of the test.

A repository composes the dataset with two folders, these are:

• Accelerometer ➔ It includes the raw data acquired from the accelerometer sensor during the test;
• Results ➔ In includes the measurement of the following variables related to sit-to-stand movement: Reaction Time; Total Time; Movement Time; Maximum Acceleration; Minimum Acceleration; Stand-Up Time; Maximum Relative Acceleration; Minimum Relative Acceleration; Maximum Velocity; Minimum Velocity; Maximum Force; Minimum Force; Maximum Power; Minimum Power; Average Velocity; Average Force; Average Power; Relative Power.

These folders include the data related to 205 experiments, where the individuals performed the sit-to-stand movement 6 times. The acceleration values are reported in m/s^2^ in the different folders. However, the velocity measurements are reported in m/s, the force values are reported in Newtons, and the power values are reported in Joules.

Considering the data reported in the different folders, Table 1 presents the statistical data related to each repetition of the Sit-to-Stand test.

**Table 1:** Statistics data of the data acquired in the different experiments.

	*N*	Minimum	Maximum	Mean	Standard deviation	Variance	Skewness	Kurtosis
**Reaction time**	1227	0.20	1.32	0.40	0.17	0.03	0.85	0.81
**Total time**	1227	2.00	4.50	2.87	0.56	0.31	0.71	0.12
**Movement time**	1227	0.97	4.29	2.47	0.58	0.33	0.71	0.34
**Maximum acceleration**	1227	10.12	87.61	18.80	7.54	56.88	3.55	17.72
**Minimum acceleration**	1227	0.12	9.61	4.69	1.96	3.84	−0.20	−0.80
**Stand-up time**	1227	0.50	14.99	2.23	1.93	3.72	4.93	26.08
**Maximum relative acceleration**	1227	0.31	77.80	8.99	7.54	56.88	3.55	17.72
**Minimum relative acceleration**	1227	−9.69	−0.20	−5.12	1.96	3.84	−0.20	−0.80
**Maximum velocity**	1227	0.00	0.26	0.02	0.02	0.00	4.80	35.24
**Minimum velocity**	1227	−0.07	0.00	−0.01	0.01	0.00	−1.71	8.85
**Maximum force**	1227	593.06	7797.27	1338.55	708.14	501466.16	3.55	17.69
**Minimum force**	1227	9.19	721.07	327.36	147.93	21884.05	0.05	−0.76
**Maximum power**	1227	0.62	1878.38	44.16	108.62	11797.35	8.79	105.42
**Minimum power**	1227	−74.94	−1.08	−4.78	3.47	12.02	−9.83	161.99
**Average velocity**	1227	0.33	10.00	2.79	0.98	0.96	1.49	11.27
**Average force**	1227	397.31	935.87	619.71	122.70	15056.38	0.53	−0.20
**Average power**	1227	168.35	7110.62	1722.03	697.54	486567.28	1.92	11.59
**Relative power**	1227	2.94	88.29	24.65	8.64	74.61	1.49	11.27

## 2 Experimental Design, Materials and Methods

### 2.1 Participants

Table 2 presents a total of 40 institutionalized older adults (20 women and 20 men) from the same institutions but living in different community-dwelling centers, volunteered to participate in the data collection (Table 1). Inclusion criteria were age ≥ 65 years old, male and female and able to stand-up from a chair with the arms crossed over the chest. Exclusion criteria were severe cognitive impairment (mini-mental state examination score < 20), cardiovascular and respiratory disorders, musculoskeletal injuries in the previous three months and terminal illness. All participants received detailed information regarding the different procedures and provided a written or oral (illiterate participants) informed consent. This acquisition of this data was approved by the Ethical Committee of the University of Beira Interior (code: CE-UBI-Pj-2019-019) and followed the recommendations of the Declaration of Helsinki.

**Table 2:** Participants characteristics.

	*n*	Age (years)^a^	Weight (kg)^a^	Height (m)^a^
**Women**	20	81.9 ± 8.1	65.4 ± 11.6	1.49 ± 0.1
**Men**	20	76.0 ± 8.2	78.0 ± 15.5	1.66 ± 0.1
**Total**	40	78.9 ± 8.6	71.7 ± 15.0	1.57 ± 0.1

a Data are mean ± standard deviation.

### 2.2 Procedure

The experimental procedures were carried out throughout ten weeks in the same place, at the same time of the day (10:00 a.m.–11:00 a.m.), at a room temperature between 22 and 24 °C. One week before, we performed a familiarization session to teach the participants the correct execution in the sit-to-stand test. During this period, we also measured the body mass and height. Then, we performed one session per week to acquire the data in the sit-to-stand test. Before every session, all participants completed a 10-min general warm-up, consisted of light walking and mobility exercises. All participants were assessed individually in a quiet place for 3 min. Before the sit-to-stand test, we placed the mobile phone inside a waistband and then attached it to the waist of the participants. After, we instructed the participants to sit on an armless chair (0.49 cm) with the back straight and the arms crossed over the chest, and to perform one repetition as fast as possible when they heard an acoustic signal emitted by the mobile application. All participants performed six repetitions, interspersed by 15-s rest. During the test, we stood alongside the participants to guarantee safety during the ascending and descending phases.

The signals are collected and stored in text readable files for further analysis.

### 2.3 Statistical analysis

The variables related to the acceleration, velocity, and power, were calculated. Considering the variables calculated and the characteristics of the sample, different descriptive statistics analysis were performed, including the calculation of mean, standard deviation, maximum, minimum, variance, Skewness and Kurtosis. The data was analyzed using mathematical and statistics software [12,13].

## 3 Ethics Statement

The participants or respective responsible person signed an ethical agreement to allow us to share the data related to the test in an anonymous form. The agreement also provided the participants’ informed consent considering the risks and the objective during the data acquisition. It was approved by the Ethical Committee of the University of Beira Interior (code: CE-UBI-Pj-2019-019) and followed the recommendations of the Declaration of Helsinki.

## Declaration of Competing Interest

The authors declare that they have no known competing financial interests or personal relationships that could have appeared to influence the work reported in this paper.

## Appendix Supplementary materials

Image, application 1
